# Could increased axial wall stress be responsible for the development of atheroma in the proximal segment of myocardial bridges?

**DOI:** 10.1186/1742-4682-4-29

**Published:** 2007-08-09

**Authors:** Pierre-André Doriot, Pierre-André Dorsaz, Jacques Noble

**Affiliations:** 1 Cardiology Department, University Hospital, Geneva, Switzerland

## Abstract

**Background:**

A recent model describing the mechanical interaction between a stenosis and the vessel wall has shown that axial wall stress can considerably increase in the region immediately proximal to the stenosis during the (forward) flow phases, so that abnormal biological processes and wall damages are likely to be induced in that region. Our objective was to examine what this model predicts when applied to myocardial bridges.

**Method:**

The model was adapted to the hemodynamic particularities of myocardial bridges and used to estimate by means of a numerical example the cyclic increase in axial wall stress in the vessel segment proximal to the bridge. The consistence of the results with reported observations on the presence of atheroma in the proximal, tunneled, and distal vessel segments of bridged coronary arteries was also examined.

**Results:**

1) Axial wall stress can markedly increase in the entrance region of the bridge during the cardiac cycle. 2) This is consistent with reported observations showing that this region is particularly prone to atherosclerosis.

**Conclusion:**

The proposed mechanical explanation of atherosclerosis in bridged coronary arteries indicates that angioplasty and other similar interventions will not stop the development of atherosclerosis at the bridge entrance and in the proximal epicardial segment if the decrease of the lumen of the tunneled segment during systole is not considerably reduced.

## Background

**Figure 1:**
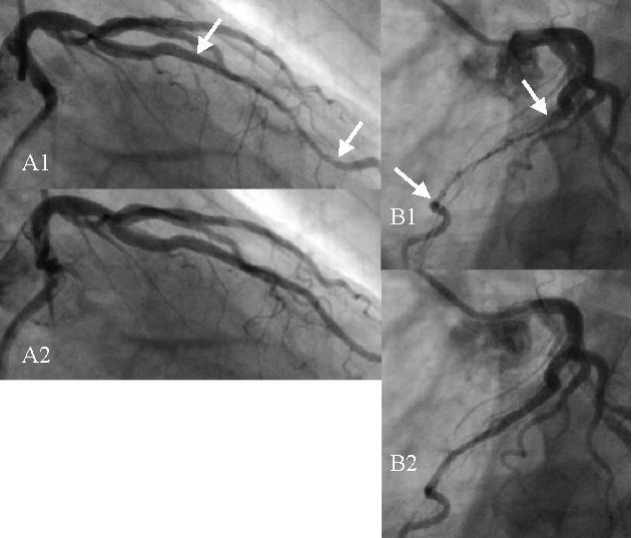
Angiographic images showing a bridge on the left anterior descending coronary artery (LAD) in a male patient of 65 years. A1) Right anterior oblique view taken at end systole. The compressed vessel segment is indicated by the two arrows. B1) Left anterior oblique view taken nearly at the same instant. A2) Same view as in A1, but taken 133 ms later. The tunneled segment is no longer compressed. B2) Same view as in B1 but 133 ms later.

The existence of myocardial bridges is known since more than a century. The interest for these anatomical particularities of coronary arteries has remained, however, very modest until the development of dynamic coronary angiography in the sixties. This new imaging modality allowed for the first time to see the compression of the tunneled vessel segment during systole ("milking effect", Fig. 1). Since that time, myocardial bridges are increasingly suspected of inducing severe ischemiae in the associated myocardial territories, and even infarcts and sudden deaths [1-5].

At necropsy, myocardial bridges are a common finding [6-8]. In the literature, the percentages vary, however, greatly but this is most probably due to differences between the definitions used by the investigators [6-9]. The left anterior descending coronary artery (LAD) is the most frequently concerned vessel [7,10,11], whereby the bridge is usually situated on the middle segment. Loukas et al. found that the presence of bridges in the adult human heart is related to coronary dominance, particularly in the left coronary circulation [12]. With angiography, the detection rate is much lower than at autopsy because only bridges having a marked compressive effect are identifiable [13-16]; it was also found that only LAD bridges are detected [17].

Bridged LAD are particularly prone to become atherosclerotic. Most authors (except for instance Edwards [7]) agree on the fact that atheroma and stenoses are frequent in the proximal adjacent vessel segment, practically inexistent in the tunneled segment, and rare in the distal one [4,9-11,14,18-23]. The reasons of this particular distribution have been studied by different authors. Ge and co-workers performed intravascular ultrasound and pressure measurements in patients and came to the conclusion that bridges augment systolic pressure and wall shear stress (WSS) in the proximal vessel segment [9,14,15]; they postulated that this induces wall damages. Möhlenkamp and co-workers thought, on the contrary, that the formation of atheroma proximal to the bridge is due to low WSS [5]. More recently, Bernhard et al. designed a mathematical-physical model to investigate the relative importance of several physical parameters involved in the hemodynamics of myocardial bridges [24]. They found that WSS and WSS oscillations are maximal in the entrance region of the bridge, and they stated that the proximal segment is more susceptible to develop atherosclerosis firstly because the pressure is increased in that segment, and secondly because WSS and WSS oscillations are maximal. With regard to the tunneled segment, they thought that it is relatively spared because WSS fades towards the end of the bridge. Concerning the distal segment, they explained that it is not exposed to the same risk because WSS is very low, and negative in the regions exhibiting flow separation; furthermore, the bridge reduces systolic pressure. Many authors assume that the tunneled segment is protected against atherosclerosis because the bridge reduces circumferential wall stress, especially during systole. This explanation is, of course, not applicable to the distal segment.

In the present contribution, we propose a different explanation for the higher susceptibility of the bridge entrance for atherosclerosis. It is based on the concept that a severe lumen reduction can generate, during the forward flow phases, a considerable increase in axial wall stress in the vessel segment situated immediately upstream of the obstruction. This concept has been described in detail elsewhere [25-27].

We begin below with a brief recall of the definitions of wall stresses. Then, we describe in a simplified manner the mechanism by which an arterial stenosis may increase axial wall stress (More detailed explanations are given in the Appendix). After a section summarizing the particularities of bridged coronary arteries, the relevance of the concept of increased axial wall stress for myocardial bridges is examined and a numerical example is calculated.

## Methods

### Definitions of wall stresses

**Figure 2:**
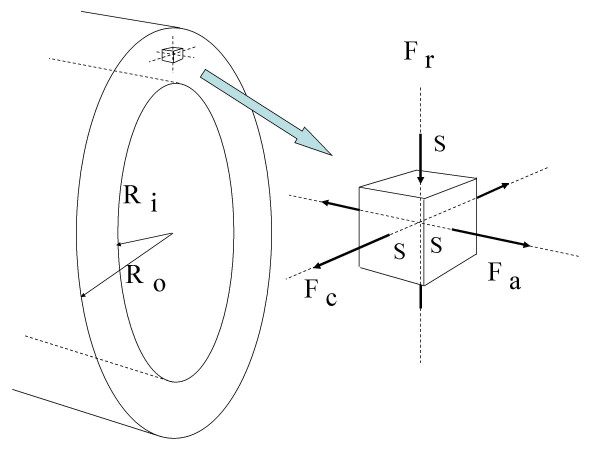
Definition of circumferential, axial, and radial wall stress (perspective view). Division of the circumferential force F_c _by the area S of the cube face it pulls at yields the circumferential wall stress σ_c _= F_c_/S. Division of the axial force F_a _by the area S of the cube face it pulls at yields the axial wall stress σ_a _= F_a_/S. Division of the radial force F_r _by the area S of the cube face it pushes on yields the radial wall stress σ_r _= F_r_/S. These three orthogonal stresses are used to describe the mechanical state of the vessel wall at the considered location. The average axial wall stress over a wall cross-section is equal to the quotient "force pulling axially at that cross-section, divided by the area A of that cross-section" (A = π (R_o_^2 ^- R_i_^2^)).

The mechanical state of a vessel at any particular location inside or on the wall is usually described by the values of circumferential, axial (also called "longitudinal"), and radial stress at that location. These stresses are defined as "force pulling perpendicularly at (or pushing perpendicularly on) the considered surface" divided by "the area of that surface" (Fig. 2). As zero-reference of stresses, one chooses usually the atmospheric pressure (A consequence of this convention is that all forces due to the compressive action of the atmospheric pressure are ignored). If the force is pulling at the considered surface, the stress is tensile, and positive by convention. If the force is pushing, the stress is compressive, and negative by convention. The three stresses are orthogonal and express the tractions the wall "material" experiences at the considered location in the circumferential and axial directions, and the compression it experiences in the radial direction. For a complete description of the mechanical state of the wall at the considered location, one needs, in principle, also the values of the circumferential, axial, and radial shearing stresses at that location; these stresses were shown, however, to be quantitatively negligible (This also holds for the well known WSS at the lumen surface of the wall). Thus, if all forces acting on and inside an excised, unloaded vessel segment are exclusively due to the atmospheric pressure, all stresses are zero by convention. In excised, unloaded vessel segments there are still small forces that are not due to the ambient pressure but to residual constraints in the "material". These forces are responsible for the well known residual stresses that can be removed by a radial cut of the vessel segment.

Blood vessels being not rigid bodies, an increase of circumferential or axial stress at a particular location is always accompanied by an elongation of the wall "material" in the corresponding direction and at that location. Thus, an increase of axial wall stress in a particular wall cross-section is always accompanied by an axial elongation of the vessel in that region.

Due to its direct relationship with the intravascular pressure, circumferential stress has always received a lot of attention, while axial and radial stresses were practically ignored. Since a few years, however, biological processes induced in arterial walls by axial stress changes are increasingly studied [28].

### Effect of a stenosis on axial wall stress

**Figure 3:**
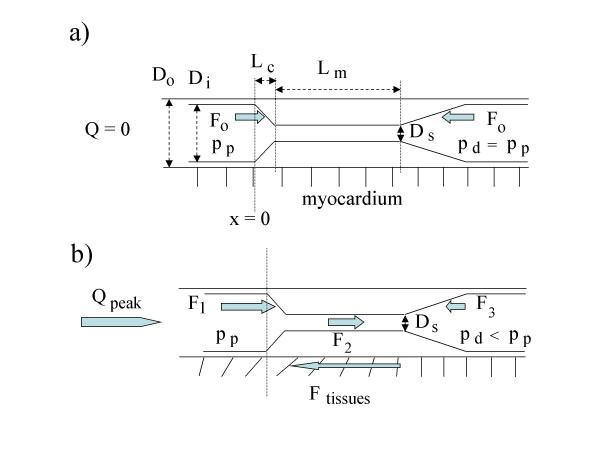
Schematic representation of a stenosed, non bridged coronary artery: a) When flow is zero, the intravascular pressure p exerts two axial, opposite, equal forces (F_o _and F_o_) in the constriction and expansion cones, respectively. The vertical equidistant slashes indicate that the vessel wall does not pull (axially) at the surrounding myocardium. b) When blood flows through the stenosis, the proximal pressure p_p _is greater than the distal pressure p_d_, and the sum of the two forces pulling in downstream direction (F_1 _and F_2_, see Appendix) is greater than the sum of the two forces pulling in upstream direction (F_3 _and F_tissues_). If flow and proximal pressure do not reach their maximum simultaneously, the net force F = F_1 _+ F_2 _- F_3 _- F_tissues _is not necessarily maximal when flow or proximal pressure are maximal. The oblique slashes show where the vessel wall will elongate axially and pull at the myocardium.

In this section, we explain in a simplified manner how stenoses may increase axial wall stress in the proximal segment during the (forward) flow phases, particularly in the segment just upstream of the entrance cone; more detailed explanations can be found in the Appendix and in references [26] and [27]. Any moderate or severe stenosis produces a decrease of the intravascular pressure in the distal segment during the flow phases, due to the pressure drop across the obstruction (Fig. 3). The magnitude of the pressure drop depends on the stenosis severity and on the instantaneous flow (among else). The difference between the pressures in the entrance and exit cones of the stenosis generate, together with the drag of the blood in the stenosis throat, an axial force. Spatially, this force is maximal in the wall cross section situated just upstream of the entrance cone (cross-section x = 0 in Fig. 3). Since the vessel is more or less tethered to the surrounding tissues, the local wall elongations induced by this force generate in turn retaining forces in these tissues. The resulting effect of all these forces is a cyclic, supplementary axial stress that is maximal in the wall cross-section x = 0. This supplementary stress adds to the "normal" axial wall stress, which is here the stress that stretches the vessel to its in vivo length, and also the axial stress one would measure in the wall during the zero-flow phase.

As the numerical examples given in reference [25] show, the supplementary stress induced in the cross section x = 0 strongly depends on the degree of stenosis (and on instantaneous flow, among else). If the stenosis is tight, this increase of axial stress may be greater than the "normal" axial wall stress of the cross-section x = 0. It is reasonable to think that arteries that do not experience axial stress variations when they are still non diseased (e.g. intact coronary arteries) are not able to resist strong, cyclic increases of axial stress without damages. In the last ten years, deleterious effects induced by axial overstretching of the vessel wall have indeed been increasingly reported (e.g. circular tears of the endothelium, or direct induction of pathologic reactive processes inside the wall).

### Particularities of bridged coronary arteries

Before examining the relevance of the mechanical model described above for bridged coronary arteries, it is necessary to recall first the morphologic and hemodynamic particularities of these arteries. They can be summarized as follows [4,5,9,14,21,29,30]. - Length and thickness of muscular bridges are quite variable. - The wall of the tunneled vessel segment is usually thinner than the walls of the proximal and distal segments; this is often due to less intima thickening, but it may also be due sometimes to a thinner media, which is perhaps a consequence that circumferential wall stress is reduced by the surrounding myocardium, particularly during systole. - During systole, the lumen of the tunneled segment is smaller than the lumens of the proximal and distal segments, and the blood velocity is greater [29]. - During diastole, the lumen of the tunneled segment often remains smaller than the lumen of the proximal segment; sometimes, it also remains smaller than the lumen of the distal segment (at least in symptomatic patients [30]). - Diastolic flow begins with a sharp flow velocity spike, which is followed by a dome-shaped pattern [9,14]; this particular picture of flow velocity is sometimes called "finger tip". The spike is due to the rapid release of the constriction inside the myocardium at early diastole when the intravascular volume of the tunneled segment is still minimal. - Antegrade systolic flow is most often reduced or absent [14]. - Retrograde flow in the proximal segment may be present during systole [29], and thus also in the tunneled segment (or in a part of it), especially after an intracoronary injection of nitroglycerin [9]. In this case, the pressure is higher in the bridge than in the proximal segment during systole [29], and it produces a transient increase of the pressure in the proximal segment. More information on the cyclic increases and decreases of pressure in the proximal, tunneled, and distal segments can be found in the article of Bernhard et al. [24].

The locations at which atheroma or stenoses are frequently encountered are usually described in the literature by "proximal to the bridge" or "on the proximal LAD segment". Some authors are more precise and specify "immediately proximal to the bridge", or "just before the bridge", or "at the entrance to the tunneled segment" [5,8,20,31,32]. A reason why the location of the lesions is not always precisely specified is probably that this point is considered to be of minor interest. For explaining the atherogeneicity of myocardial bridges it is, however, important. Anyway, several other observations confirm this particularity of bridges. For instance, Polacek found intima thickening in the segment immediately proximal to the bridge (and sometimes also behind the bridge) [8]. Similarly, Ishii et al. observed that the ratio "intima thickness to media thickness" is higher immediately before the bridge than at any other site when the bridge is situated on the proximal LAD segment [31]. Boucek et al. used the level of incorporation of ^35^SO_4 _into glycosaminoglycans (GAG) to identify the sites of accentuated stress in coronary arteries of dogs; they found that the metabolism of GAG was higher in the epicardial segments, particularly in the segment immediately proximal to the bridge [33].

Concerning the severity of the atherosclerotic lesions, Ge et al. have pointed out that proximal stenoses can be quite important (mean area stenosis: 45%) [9]. Bridged coronary arteries can also be "angiographically normal" at end-diastole [1,34] but this does not mean, of course, that they are non diseased.

### Proposed explanation for the atherogeneicity of myocardial bridges

Based on the facts mentioned in the preceding sections and on further considerations that will be developed in this section, our proposition is that cyclically excessive axial wall stress at the bridge entrance is responsible (at least partly) for the great susceptibility of bridged coronary arteries for atherosclerotic degradations in this region.

A first, evident case in which axial wall stress cyclically increases in the proximal segment is, of course, that the tunneled segment pulls axially at that segment during systole, due to strong morphologic changes in the bridge region during the heart contraction (e.g., deeper dipping of the tunneled segment into the myocardium during systole). Atherosclerotic degradations may then be expected at the entrance of the bridge, and possibly further upstream of the entrance. If the distal segment also experiences such a cyclic pulling, degradations may also be expected at the bridge exit.

A second, less obvious possibility for cyclic increases of axial wall stress originates in the hemodynamic changes that occur in bridged coronary arteries during the cardiac cycle. Basically, the mechanical model used in the present study predicts a cyclic increase of axial wall stress there where the flowing blood encounters a severe lumen reduction. Since bridged arteries are not quite comparable to coronary arteries with a permanent stenosis, we have to consider two cases. It is assumed that cyclic morphologic changes in the previously mentioned sense are negligible in both cases.

Case 1) In this first case, the lumen of the tunneled segment shall be smaller during the whole cardiac cycle than the lumen of the proximal adjacent segment [29,34]. As soon as blood flows (forward), an axial force F appears, due to the unbalance between the axial force F_1 _+ F_2 _pulling in downstream direction (see Fig. 3b) and the axial force F_3 _+ F_tissues _opposing the force F_1 _+ F_2 _(F = F_1 _+ F_2 _- F_3 _- F_tissues_; see Fig. 3b and Appendix). The force F_1 _is due to the pressure pushing in the entrance cone of the bridge; F_2 _is the force generated by the drag of the blood in the tunneled segment; F_3 _is due to the pressure pushing in upstream direction in the exit cone of the bridge, and F_tissue _is the retaining axial force provided by the myocardial tissues surrounding the artery distally of the cross section x = 0. In the following, we mainly consider the effect of these four forces in the wall cross section x = 0. In that cross section, the force F = F_1 _+ F_2 _- F_3 _- F_tissues _produces a cyclic increase of axial wall stress (This effect is, of course, not limited to the cross section x = 0; it is in fact present in all cross sections situated upstream of the exit cone of the bridge, but with attenuated magnitude because "abnormal" axial wall forces are transmitted to the surrounding myocardial tissues via the axial displacements/elongations of the vessel they induce. For instance, if the proximal segment were not tethered to the myocardium, the force F would be present with full magnitude in that segment).

The spatial maximum of the force F is always in the cross section x = 0. Of great importance is further the magnitude of the temporal maximum of F, because the supplementary axial wall stress generated in the entrance region of the bridge and the resulting axial wall stretch are proportional to F. At which precise time point the force F reaches its maximum is, in se, not important. It may be when the contracting myocardium abruptly reduces diastolic flow (early systole), or rather at the time of the "finger tip" (flow velocity spike at early diastole), or at some other time during the flow velocity "dome". The determination of this time point would require the use of a sophisticated hemodynamic model for bridged LAD arteries.

Case 2) In this second case, the lumen of the tunneled segment during diastole shall be nearly equal to the lumen of the proximal segment. The force F present in the wall just upstream of the bridge entrance is perhaps maximal at early systole when the contracting myocardium abruptly reduces the lumen of the tunneled segment, but it is also conceivable that F is maximal at the time of the "finger tip" (provided that such a flow velocity spike is present), or at a particular time point of the "dome" phase.

In bridged coronary arteries, the lumen of the distal (epicardial) segment remains usually at least equal to the lumen of the tunneled segment throughout the cardiac cycle. Thus, the flow never encounters a decrease of the lumen area at the bridge exit. As a consequence, there is never a cyclic increase in axial wall stress due to flow, and the development of atheroma should therefore not be promoted at this site.

With regard to the well known risk factors of atherosclerosis (hypertension, diabetes, hypercholesterolemia, etc), it seems obvious that these factors play the same aggravating role as in non bridged arteries.

### Numerical example

To qualitatively illustrate the effect a bridge may have on axial wall stress in the entrance region of the bridge, we consider a 3 mm (lumen diameter) LAD with a 12 mm bridge at the end of the first segment. The vessel diameter of the tunneled segment during diastole shall be 3 mm. In order to calculate the pressure drop across the bridge as a function of the percent diameter reduction (DS) jointly defined by the tunneled segment and the proximal segment, we have to choose values for the proximal pressure and the flow. According to a study of Ge et al. [14], myocardium contraction begins to reduce the end-diastolic flow through the bridge practically at the time of maximum aortic pressure. For the pressure at the bridge entrance (proximal pressure), which is nearly equal to aortic pressure, we choose therefore 120 mmHg. Based on the same study we assume next an instantaneous flow velocity of 7 cm/s in the proximal segment (average value over the lumen). This yields an instantaneous flow of 1 ml/s (These choices do not imply that the force F is necessarily maximal at the time of aortic maximum pressure). Using the formulas given in [25], we calculate then the pressure drop across the bridge for the DS range 1% to 99%. In the computer algorithm, flow is automatically reduced at high DS values in such a way that the distal pressure does not fall below a chosen limit (see Appendix). The rationale of this is that in case of severe (conventional) stenosis a minimal diastolic distal pressure of about 10 mmHg is needed to push some blood through the fully dilated arterioles and the capillary bed. Although this value may be less founded for bridges, we use it as lowest limit for the pressure at the bridge exit. Thus, the algorithm reduces the chosen flow value (1 ml/s) at high DS values to a level such that the pressure drop across the bridge does not exceed the proximal pressure minus 10 mmHg.

For the computation of the axial force F that pulls at the cross section x = 0 at the DS values 1% to 99% (see Fig. 3), we assume for simplicity (and arbitrarily) that the drag force F_2 _that pulls at the inner surface of the tunneled segment is exactly compensated by the retaining axial force F_tissues _provided by the surrounding tissues downstream of the bridge entrance (see Appendix). Since the bridge is rather short (12 mm) and the flow reduced at high DS values, this simplification has not a great impact. The supplementary axial force F that pulls cyclically in the vessel wall just ahead of the bridge entrance is thus exclusively due to the forces F_1 _and F_3 _exerted by the blood onto the inner surfaces of the constriction and expansion cones: F = F_1 _- F_3 _(see Appendix). Having calculated the values of F for the DS range 1% to 99%, we assume then a relative wall thickness t_r _of 1.15 at the cross section x = 0 (t_r _= D_o_/D_i_, where D_i _= 3 mm and D_o _= D_i _t_r _= 3.45 mm are the inner and outer diameters of the proximal LAD segment at x = 0) and calculate the resulting supplementary axial stress values at x = 0 as F/[0.25 π (D_o_^2 ^- D_i_^2^)].

**Figure 4:**
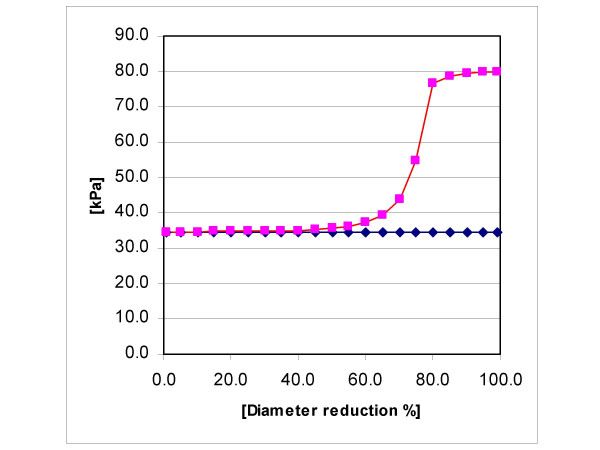
Axial wall stress (y-axis) at the entrance of the bridge considered in the numerical example versus diameter reduction values (DS; x-axis). The stress values are the sum of "normal" axial wall stress (see text) and supplementary axial stress generated cyclically by the pressure drop across the bridge. The flow was set to 1 ml/s as long as the distal pressure did not fall below 10 mmHg. At high DS values (80, 85, 90, and 99%), it was appropriately reduced in order to respect this 10 mmHg limit. Axial stress begins to increase markedly at a DS value of approximately 60%; this corresponds to a lumen area reduction of roughly 80%.

In order to obtain the value of axial wall stress at x = 0 at a particular DS value, we have to add the corresponding, supplementary axial wall stress generated by the force F to the "normal" axial stress of the proximal LAD segment. This "normal" axial stress is the stress needed to stretch the artery to its in vivo length. In epicardial coronary arteries of adults (except perhaps in aged individuals), it can be assumed to have roughly the value one would reach by inflating an excised, occluded segment of the considered artery with a pressure equal to the mean in vivo intravascular pressure [25]. Choosing 84 mmHg for this mean value, we obtain, with D_i _= 3 mm and t_r _= 1.15, a "normal" stress value of 34.6 kPa. Adding this "normal" axial wall stress to the computed values of the supplementary axial stress generated by the force F yields the curve depicted in Fig. 4. The flattening of the curve at high DS values (80, 85, 90, 95, and 99% in this example) is due to the previously mentioned flow reduction at high DS values, but not to the chosen limit of 10 mmHg (Setting 0 mmHg instead of 10 mmHg did not markedly modify the curve).

As Fig. 4 shows, axial wall stress at the bridge entrance does not increase appreciably as long as DS < 60%, but at values greater than 80% it has more than doubled. Since increases in axial wall stress and resulting axial elongations are proportional (in first approximation), a stress increase of 100% results in a local axial elongation of roughly 100%. For comparison, if one assumes for simplicity that the diameter of a coronary artery does not greatly increase if systolic pressure doubles, a 100% increase of circumferential stress corresponds roughly to an increase of systolic pressure from, for instance, 140 mmHg to 280 mmHg. Since coronary arteries are not primarily structured to cope with great variations of axial wall stress, it is likely that increases of axial stress of 20% or more at the bridge entrance might be the main reason why this region often exhibits atheroma, or even a conventional stenosis. It must be underlined, however, that this result does not allow to exclude high or low WSS at the bridge entrance from the culprit list [cf. [24]].

Since the first segment of the LAD is rather straight and not very rigidly attached to the myocardium, it is conceivable that the axial force generated by the bridge is not very efficiently absorbed by the myocardial tissues. As a consequence, the cyclic increase in axial wall stress present in the cross section x = 0 may also be present farther upstream, although with attenuated magnitude. This might explain why the segment upstream of the bridge is more prone to become atherosclerotic than the same segments of non bridged arteries. Similarly, the fact that the supplementary axial force generated in the wall upstream of the bridge entrance decreases with increasing upstream distance from the bridge entrance might explain why the first segment of a bridged LAD artery is more prone to become atherosclerotic when the bridge is situated at the end of this segment than when it is situated at the end of the second or third segment.

## Results and Discussion

The explanation proposed in this article for the fact that in bridged coronary arteries atherosclerosis develops mainly at the bridge entrance is based on the concept that axial wall stress becomes cyclically excessive at this site, and that this abnormal stress induces wall damages. The underlying postulate that cyclic axial overloads are less well tolerated than the cyclic increases in circumferential stress generated by the normal pressure pulses is based on the fact that the structure of arterial walls is mainly circumferential, and that the axial sections of the vessel are less coupled. The effect of axial wall stress is therefore of much more interest for wall damages than circumferential stress, which is present during the cardiac cycle.

In vessel modeling one has to simplify many things and to make assumptions. The numerical results presented in the preceding section can therefore not be accurate to a few percents. Nevertheless, they clearly indicate that axial wall stress may considerably augment in the segment immediately proximal to the bridge entrance in the course of each cardiac cycle. Due to the variability of the many parameters involved (pressures, flow, bridge morphology, compression strength, etc), the cyclic stress increase exhibits most probably a considerable inter-individual variability; in some bridges, it will perhaps be greater than 100% while in other ones it will be much less. Furthermore, the magnitude of axial wall stress, and of increases of this stress, along a vessel segment also depends on the action of the tethering forces exerted by the surrounding tissues. But the increase in axial stress will be maximal at the bridge entrance because, further upstream, the cyclic axial wall force F is progressively absorbed by the surrounding tissues.

According to Fig. 4, axial wall stress becomes clearly excessive only at high DS values. As a consequence, the proximal segment of bridged arteries in which the lumen of the tunneled segment is not strongly reduced during systole should exhibit less atherosclerosis than the proximal segment of bridged arteries in which the tunneled segment undergoes a strong compression.

The proposed concept of excessive axial stress does not apply to the tunneled segment itself. For this segment, one can make the following considerations. If the tunneled segment is firmly attached to the myocardium, axial forces of hemodynamic origin cannot induce appreciable cyclic variations of its length. If the axial forces generated by the deformation of the myocardium in the region of the bridge are, moreover, also negligible, then axial wall stress remains constant, irrespectively of the actual wall thickness of the segment. Thus, atherosclerotic modifications of the wall, if any, should not be due in this case to excessive variations of axial wall stress. This is independent of the actual value of axial stress, which may be lower, equal, or higher than in the proximal and distal epicardial segments. The actual stress value cannot be predicted; one can presume, at most, that it is comparable to the "normal" axial wall stress of the proximal and distal segments.

If the tethering forces acting axially on the tunneled segment are, on the contrary, negligible, as it is possibly the case when the segment is embedded in a thick layer of fat and thus not firmly attached to the myocardium, then the axial wall stress of the tunneled segment will be greater than in the proximal and distal epicardial segments if the diastolic lumen area of the tunneled segment is equal to (or smaller than) the lumen area of the proximal and distal segments, and the wall thinner (Thereby, constant length of the tunneled segment and wall incompressibility are assumed). But this difference in axial stress will be permanent because it is due to the smaller lumen and/or the smaller wall thickness of the tunneled segment. Since the SMC inside the wall of the tunneled segment are in this case not submitted to axial wall elongations induced by cyclic increases of axial stress, this permanent stress difference has (presumably) no deleterious effects Pathologic modifications that are exceptionally found well inside the tunneled segment [5,7] may therefore be due to excessive shear stress inside this segment during systole or early diastole, or to a cyclic elongation of this segment due to morphologic changes in the bridge region.

If there is a lumen reduction at the bridge exit during diastole (which does not mean that such cases really exist), and if the tunneled segment is embedded in a thick layer of fat, then a cyclic elongation of the arterial wall of the tunneled segment is possible, particularly just proximal to the bridge exit. This prediction is consistent with the fact that pathologic wall modifications are more frequent when the fat layer around the tunneled vessel segment is thick [11].

The concept of cyclically excessive axial stress appears to be also consistent with results published by different authors. For instance, Ishikawa et al. studied 108 rabbits fed with a cholesterol diet (ChoR) and 29 control rabbits (ConR) [23]. In the rabbits they used, a part of the LAD is always tunneled. Groups of ChoR were sacrificed at 1 week intervals up to the 20th week, and groups of ConR were sacrificed after 1, 8, and 20 weeks. The last 3 mm segment immediately proximal to the tunneled LAD (called EpiLAD) and the first 3 mm of the tunneled segment (MyoLAD) were examined. The tunneled segment appeared to be still normal in the ChoR and the ConR. The EpiLAD of the ConR were also normal but 1A4 (alpha smooth muscle actin, Dakopatts, Denmark) was found in the cytoplasm of smooth muscle cells of the media. In the EpiLAD of the ChoR, raised lesions grew very rapidly after the 10th week. If one considers that cholesterol played in that study the role of a "marker" of favorable conditions for atherosclerosis, then the results show that such conditions are totally absent in tunneled segments but fulfilled in the EpiLAD of the ChoR, and probably in the EpiLAD of the ConR, too. Since the endothelial cells had different shapes in the MyoLAD and the EpiLAD, Ishigawa attributed the different behavior of MyoLAD and EpiLAD to shear stress differences. However, one can as well come to the conclusion that the observed differences were due to excessive axial wall stress in the arterial segment immediately proximal to the tunneled segment. One can, of course, not exclude that also circumferential stress increased too much during early systole. Distal segments were not examined. Boucek and co-authors found that the metabolism of glycoaminoglycan (GAG) is much higher in the segment immediately proximal to the bridge than in the tunneled segment [33]. This is also an important observation because it shows that increased GAG metabolism is indeed found there where increased axial stresses can be expected. Of note is, moreover, that they attributed this phenomenon to axial stress, which is quite unusual in the literature about atherosclerosis. Their findings are thus in agreement with our concept. The same applies to the results of Polacek who found intima thickening in the segment immediately proximal to the bridge [8].

A further observation that supports the concept of excessive axial stress is that atheroma at the bridge entrance is more severe when the fat layer between myocardium and tunneled segment is thick [11]. This observation is easily explainable by the fact that in this case the force F_tissues _(see Appendix) is weaker.

Like non bridged coronary arteries, bridged ones can be angiographically normal during diastole [34]. This does not prove, however, that they are free of atherosclerosis because uniform intima thickening is seldom detectable angiographically. Inversely, our concept does not exclude that some bridged arteries may be non diseased. This might be the case for instance when there is no great diameter differences between epicardial and tunneled segments during diastole.

As previously mentioned, cyclic increases of axial wall stress may also be due to morphologic changes in the bridge region. The tortuosities observed by Klues et al. and Channer et al. [29,35] at bridge entries or exits during diastole may be a consequence of such a cyclic axial pulling at the tunneled segment.

The mechanical model used in the present contribution was originally developed for conventional stenoses affecting conductance or distribution arteries [25]. It was shown later to be consistent with published observations about radioactive stents, catheter-based brachytherapy, and conventional stents [36,37]. The explanation of atherosclerosis in bridged coronary arteries proposed in this article is quite different from the one proposed by Ge and coauthors [15] who suggested increased circumferential and WSS as probable reasons. It is also different from the ones of Klues and coauthors [29] and of Bernhard and coauthors [24] who also incriminated WSS. It must be underlined, however, that our concept cannot invalidate these different explanations (and inversely). In fact, it is quite compatible with these explanations. It is also possible that excessive axial wall stress and WSS have a combined causal action. On the other hand, the concept of excessive axial wall stress provides also an explanation for the fact that the intensity of atherosclerotic developments in the proximal LAD segment is greater when the fat layer between tunneled segment and myocardium is thick [11] or when the bridge is situated on the upper segment of the LAD [4,11,30]. This fact may not be easily explainable by excessive shear or circumferential stresses [15,29].

## Conclusion

Cyclically excessive axial wall stress at the entrance of myocardial bridges appears to be a possible explanation for the great susceptibility of this site to become atherosclerotic. With regard to clinical implications, the proposed explanation suggests that reduction or suppression of a (conventional) stenosis at the bridge entrance by angioplasty, followed or not by stenting, may temporally reduce ischemia but not solve the problem once for all if the underlying cause of the atherosclerotic evolution subsists, which is the cyclic diameter reduction of the tunneled segment. This view may not be shared by all cardiologists [38,39] because one can object that the stent is implanted in such a manner as to cover also the whole tunneled segment. But stenting of bridges was shown to be associated with high restenosis rates [5,40-42]. Thus, it might turn out in the future that only surgery can suppress both ischemia and the progression of atherosclerosis in the proximal epicardial segment.

## Appendix

The mechanism by which arterial stenoses may increase axial wall stress in the segment immediately proximal to the constriction cone has been described elsewhere [25-27]. It can be summarized as follows. In a stenosed vessel (see Fig. 3), the blood exerts forces onto the inner surfaces of the constriction and expansion cones, and in the throat of the stenosis. We consider only the axial components of these forces. When flow is zero, the two forces pushing in the cones (F_o _and F_o_, see Fig. 3a) compensate each other and there is also no drag in the stenosis throat. The net force F generated in the wall cross section of interest (x = 0) is thus zero. During the (forward) flow phases (Fig. 3b), the situation is different. Because of the pressure drop across the stenosis the axial force exerted on the inner surface of the constriction cone (F_1_) is greater than the force exerted on the expansion cone (F_3_). Furthermore, a force F_2 _due to the drag of the blood in the stenosis throat pulls at the vessel in downstream direction. Most often, there is also a fourth force, F_tissues_, which is the retaining force opposed by the tissues surrounding the vessel downstream of the cross section x = 0 to axial displacements of the vessel wall with respect to the myocardium (see Fig. 3b). The supplementary axial force appearing cyclically in the wall cross section x = 0 is thus: F = F_1 _+ F_2 _- F_3 _- F_tissues_. This force is, of course, time varying but it has always its spatial maxima in the cross section x = 0.

The forces F_1 _and F_2 _increase with the degree of stenosis, the proximal pressure, and the flow rate (among else). The force F_3 _increases first with stenosis severity but decreases then when the pressure drop across the constriction becomes important.

In stenosed, non bridged coronary arteries, the force F = F_1 _+ F_2 _- F_3 _- F_tissues _is zero at zero-flow, and high at peak flow (diastole). The maximum value reached by the force F_1 _+ F_2 _- F_3 _during the cardiac cycle can be estimated in the way described in [26] by using hydraulic formulas provided by Back et al. [43]. Effects due to the cyclic movement of the heart, as studied by Moore et al. for instance [44], are not included in the model.

In bridged arteries, which are arteries with a variable "stenosis", the situation is somewhat different because it is not possible to say when the force F reaches its maximum without the help of a dynamic model. If one assumes instantaneous values for flow, pressure, and diameter reduction percent (DS), the force F_1 _+ F_2 _- F_3 _can nevertheless be estimated in the same manner as for constant stenoses. In the numerical example given in the text, we have assumed a flow of 1 ml/s and a proximal pressure of 120 mmHg. We have then calculated the force F for a DS range of 1% to 99%, under the simplifying assumption that F_2 _was exactly compensated by F_tissues_, irrespective of the actual DS value.

Since imposing a proximal pressure and a flow can result, at high DS values, in a calculated pressure drop that exceeds the proximal pressure (which is, of course, impossible), it is necessary in these cases to reduce the chosen flow value appropriately. This can be done as follows. If we sum algebraically the different equations that yield the different pressure drops and pressure recovery occurring across the stenosis, we obtain the total pressure drop on one side, and a function of Q, Q^2^, DS, and other parameters on the other side. We have then simply to impose the maximally admissible pressure drop (e.g., p_proximal_, or p_proximal _- 10 mmHg), and to solve for Q. If the obtained flow value is smaller than the chosen one (1 ml/s in our example), it is used in place of the chosen one for the computation of the forces F_1_, F_2_, and F_3_.

If the force F is not transmitted to tissues surrounding the vessel upstream of the stenosis (see Fig. 3b), then it will be present with full magnitude in the proximal vessel segment up to the region where it can be transmitted. The transmission can, of course, also be partial; in this case the supplementary axial force present in the wall decreases with increasing upstream distance from the stenosis.

Division of the supplementary axial force cyclically generated by the obstruction at a particular axial location by the area of the wall cross-section at that location yields the supplementary axial wall stress at that location. This supplementary, cyclic wall stress adds to the (practically constant) "normal" axial stress of the vessel wall. In an epicardial coronary artery (as in many other conductance arteries with constant length), the "normal" stress can be assumed to have roughly the value one would obtain by inflating an excised segment of the artery with a pressure equal to the mean in vivo pressure at rest. It is thus practically equal to the axial stress in situ or in vivo, whereby the vessels of interest here are assumed to have no tone variation capabilities in axial direction so that "normal" axial stress and the length of the considered arterial segments are temporally constant.

## Competing interests

The author(s) declare that they have no competing interests.

## Authors' contributions

PA Doriot and PA Dorsaz designed the mathematical-physical parts of the study. J Noble worked out the medical aspects. All authors have read and approved the final manuscript.
